# Yeast Model Uncovers Dual Roles of Mitochondria in the Action of Artemisinin 

**DOI:** 10.1371/journal.pgen.0010036

**Published:** 2005-09-16

**Authors:** Wei Li, Weike Mo, Dan Shen, Libo Sun, Juan Wang, Shan Lu, Jane M Gitschier, Bing Zhou

**Affiliations:** 1 The State Key Laboratory of Biomembrane and Membrane Biotechnology, Department of Biological Sciences and Biotechnology, Tsinghua University, Beijing, China; 2 Departments of Medicine and Pediatrics and Howard Hughes Medical Institute, University of California, San Francisco, California, United States of America; Washington University, United States of America

## Abstract

Artemisinins, derived from the wormwood herb *Artemisia annua,* are the most potent antimalarial drugs currently available. Despite extensive research, the exact mode of action of artemisinins has not been established. Here we use yeast, *Saccharamyces cerevisiae,* to probe the core working mechanism of this class of antimalarial agents. We demonstrate that artemisinin's inhibitory effect is mediated by disrupting the normal function of mitochondria through depolarizing their membrane potential. Moreover, in a genetic study, we identify the electron transport chain as an important player in artemisinin's action: Deletion of *NDE1* or *NDI1,* which encode mitochondrial NADH dehydrogenases, confers resistance to artemisinin, whereas overexpression of *NDE1* or *NDI1* dramatically increases sensitivity to artemisinin. Mutations or environmental conditions that affect electron transport also alter host's sensitivity to artemisinin. Sensitivity is partially restored when the *Plasmodium falciparum NDI1* ortholog is expressed in yeast *ndi1* strain. Finally, we showed that artemisinin's inhibitory effect is mediated by reactive oxygen species. Our results demonstrate that artemisinin's effect is primarily mediated through disruption of membrane potential by its interaction with the electron transport chain, resulting in dysfunctional mitochondria. We propose a dual role of mitochondria played during the action of artemisinin: the electron transport chain stimulates artemisinin's effect, most likely by activating it, and the mitochondria are subsequently damaged by the locally generated free radicals.

## Synopsis

Malaria kills at least 1 million people worldwide a year. Recent years saw the rapid emergence of drug-resistant malaria strains. Artemisinins, derived from the Chinese wormwood herb *Artemisia annua,* are the most potent antimalarials currently available. Despite extensive research, the exact mode of action of artemisinins has not been established. In this article, Li et al. investigated yeast as a model to probe the core working mechanism of this class of antimalarials. They showed that artemisinin can disrupt the normal function of mitochondria by depolarizing its membrane potential, and that artemisinin's effect can be affected by its interaction with the mitochondrial electron transport chain, an apparatus that couples oxygen oxidation and energy generation in the cell. They proposed a dual role of mitochondria played during the action of artemisinin: the electron transport chain likely activates artemisinin, and the mitochondria are subsequently damaged by the locally generated free radicals associated with this activation. The research has provided a fine tool for the study of the mechanism of artemisinin in a model organism (yeast), and laid the framework for a set of possible future experiments to be conducted in yeast and malaria parasites.

## Introduction

Malaria, the most prevalent and pernicious parasitic disease of humans, is estimated to kill between 1 million and 2 million people, mainly children, each year. Resistance has emerged to all classes of antimalarial drugs except artemisinin, an endoperoxide antimalarial drug derived from an ancient Chinese herbal remedy, qinghaosu. The mechanism of the action of artemisinin remains a mystery, although iron appears to be involved in activating this endoperoxide to generate cytotoxic free radicals [1]. Several candidates have been hypothesized as targets of artemisinins, including haem, a translationally controlled tumor protein and some parasite membrane proteins [1–3], but none of these have been convincingly shown to be functionally relevant. Recently, Eckstein-Ludwig et al. [4] proposed PfATP6, a sarco/endoplasmic reticulum Ca^2+^-ATPase, as artemisinin's target and inferred that artemisinin might act by mobilizing intracellular Ca^2+^ stores; however, this conclusion has also been debated [5].

One important reason for the lack of satisfying progress in understanding artemisinin is that it is difficult to carry out genetic analysis of malaria parasites. In contrast, the yeast *Saccharamyces cerevisiae* is an ideal model organism to uncover a variety of molecular mechanisms that otherwise might be difficult to address with other systems. In this study, we developed a yeast model and used it to probe the fundamental mechanisms of artemisinin's action.

## Results

### Artemisinin Inhibits Yeast Growth in Nonfermentable Media

In a pilot experiment, we tested whether artemisinin is toxic to the growth of yeast cells. As demonstrated in Figure 1A, the inhibitory effect of artemisinin on yeast growth in YPD (a fermentable medium with dextrose as the carbon source) liquid was not detectable at 8 μM, and only started to be observable at 80 μM, a concentration far above the level needed to inhibit the growth of *Plasmodium falciparum* (a 50% growth inhibition concentration [IC_50_] of approximately a few nM) [6,7]. On YPD plates, no significant inhibition of growth is even noticeable in the presence of 80 μM artemisinin.

**Figure 1:**
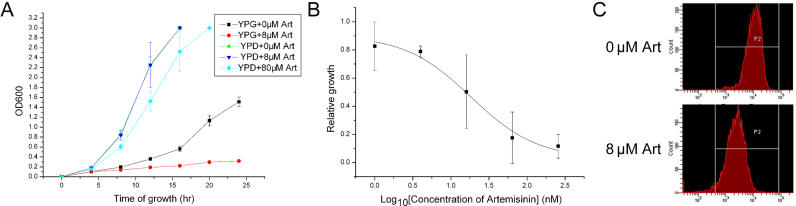
Artemisinin Inhibits Yeast Respiratory Growth by Depolarizing the Mitochondrial Membrane (A) Artemisinin (Art) inhibits yeast growth in nonfermentable media. In YPD the effect of artemisinin is minimal, whereas in YPG, artemisinin is highly effective. (B) Yeast growth is inhibited by artemisinin in YPG with an IC_50_ that is comparable to that required to kill cultured malaria parasites. Relative growth in the presence of artemisinin was measured against to that of the yeast grown in the absence of artemisinin. Experiments shown were performed three times in liquid YPG media. Error bars represent standard errors of the mean for each assay. (C) Artemisinin depolarizes mitochondrial membrane. The peak shift toward the left represents a decrease of fluorescence signal indicating the loss of membrane potential. Cells were grown in YPG with or without artemisinin (Art) for 2 h.

This level of insensitivity to artemisinin led us to consider whether use of the YPD media precluded our assessment of one important organelle, the mitochondrion, in the action of artimisinin. Yeast can grow either anaerobically or aerobically by utilizing fermentable or nonfermentable carbon sources. In the presence of a fermentable carbon source such as glucose (dextrose), as in YPD, yeast preferentially adopt glycolysis to generate energy even under aerobic conditions, and can grow normally even when mitochondrial respiration level is minimal. In order to determine whether artemisinin interferes with mitochondrial function, yeast was grown in YPG or YPE media in which glucose was replaced with the nonfermentable carbon source glycerol or ethanol. Amazingly, in YPG(E) media, yeast growth was severely inhibited when artemisinin was present. Figure 1A shows the dramatic reduction of cell growth in the presence of 8 μM. Indeed, for yeast grown in YPG, the IC_50_ (defined here as 50% growth inhibition in 48 h) was determined to be approximately 10 nM (Figure 1B), a concentration comparable to what is effective in *P. falciparum*. The dramatic contrast of sensitivity between yeast grown on artemisinin-supplemented YPD and YPG(E) media indicates that artemisinin affects mitochondrial function in YPG(E).

### Artemisinin Depolarizes the Mitochondrial Membrane

The observation of growth inhibition of yeast on artemisinin-supplemented YPG(E) media prompted us to investigate whether the mitochondrial membrane potential of yeast is affected by artemisinin, because maintenance of the membrane potential is key to mitochondrial functions, including oxidative phosphorylation and metabolism of amino acids, lipids, and haem, as well as intracellular Ca^2+^ homeostasis. As shown in Figure 1C, treatment of yeast with artemisinin caused substantial loss of membrane potential, as evidenced by the decreased rodamine uptake into the mitochondria. This loss of membrane potential was also observed with yeast grown in YPD media, but required the presence of a slightly higher concentration of artemisinin or a longer treatment time.

### Genetic Studies Reveal Electron Transport Chain Plays Important Roles in the Action of Artemisinin

To gain further insights into the mechanism of action of artemisinin, we screened for yeast genes that, when inactivated, can confer artemisinin resistance. A library comprised of 4,757 homozygous deletion strains [8,9] was plated on YPG(E) agar plates with 3–5 μM of artemisinin. Three genes whose deletion resulted in artemisinin resistance were identified. Two of these, *NDE1* and *NDI1,* encode yeast NADH dehydrogenases in the mitochondrial electron transport chain [10–12]. *ndi1Δ* and *nde1Δ* displayed similar level of growth on artemisinin-supplemented YPGE plates (Figure 2A). Overexpression of *NDE1* and *NDI1* in their corresponding deletion strains or in the parental BY4742 background (wild type) resulted in significantly increased sensitivity to the drug (Figure 2B, first three rows). These data suggest that NADH dehydrogenase activity is positively correlated with yeast sensitivity to artemisinin.

**Figure 2:**
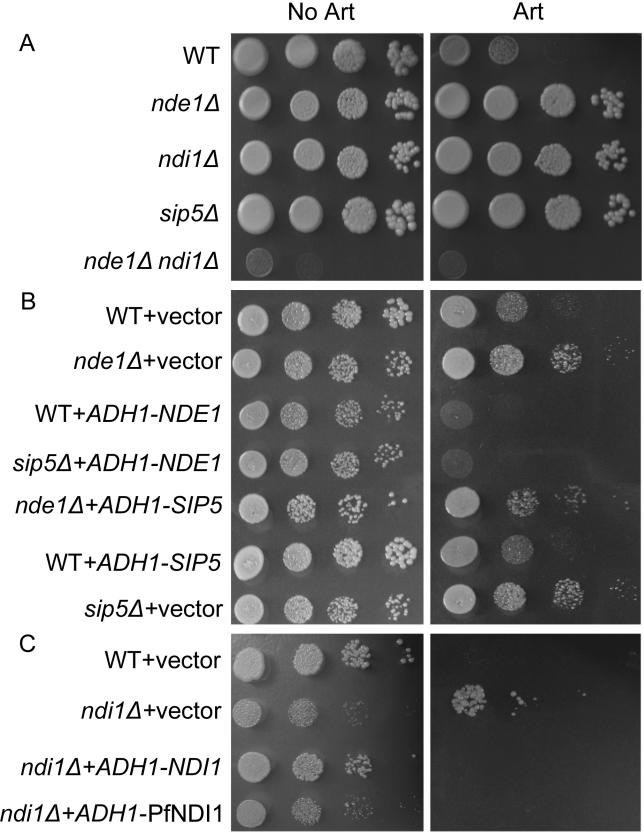
The Genetic Screen for Artemisinin-Resistant Mutations Identified Genes in the Electron Transport Chain or in the Pathway of Respiratory Control (A) The three mutants isolated display increased resistance to artemisinin. YPGE plates with or without 4 μM artemisinin were used. *nde1Δ ndi1Δ* exhibited severe growth defect in nonfermentable media. (B) Increased activities of NADH dehydrogenases exacerbate artemisinin sensitivity, and Sip5 may be positioned upstream of NADH dehydrogenases. Plates are all SG-Ura (with or without 4 μM artemisinin) to prevent plasmid loss. *ADH1-NDE1* and *ADH1-SIP5* here denote constructs that express *NDE1* and *SIP5* under the control of *ADH1* promoter. The results of *ADH1-NDI1* are similar to that of *ADH1-NDE1* and are not shown on the two plates*.* (C) Expression of PfNDI1 in *ndi1*Δ restores yeast sensitivity to artemisinin. Plates used here are SG-Ura (with or without 8 μM artemisinin). Art, artemisinin; SG, synthetic yeast media with glycerol as the carbon source; WT, wild type.

In addition to *NDE1* and *NDI1*, a third homologous yeast gene, *NDE2,* is known to provide NADH dehydrogenase activity in yeast. These NADH dehydrogenases oxidize NADH at either the cytosolic side (Nde1p and Nde2p) or the matrix side (Ndi1p) of the inner mitochondrial membrane. Although deletion of *NDE1* leads to a major loss of NADH dehydrogenase activity at the cytosolic side, deletion of both *NDE1* and *NDE2* results in complete loss of NADH dehydrogenase activity at the cytosolic side [11–13]. Because of sequence and function similarity between Nde1p, Ndi1p, and Nde2p, we asked whether *NDE2* interacts with artemisinin. Compared to that of *nde1Δ* or *ndi1Δ*, *nde2Δ* manifested only marginal resistance to artemisinin, and the double mutant *nde1Δ nde2Δ* was not substantially more resistant to artemisinin than *nde1Δ*. This is consistent with the observation that at the cytosolic side Nde1p is the major NADH dehydrogenase and Nde2p only plays a minor role [10–12].

Next, we investigated whether the double mutant *nde1Δ ndi1Δ* is more resistant to the action of artemisinin or not. *nde1Δ ndi1Δ* yeast can grow well in glucose media. However, their growth on nonfermentable media is greatly affected (see Figure 2A), indicating severely compromised mitochondrial function of the strain. This growth defect precludes our comparative analysis of *nde1Δ ndi1Δ* resistance against artemisinin.

Although three NADH dehydrogenases are present in yeast, only one such dehydrogenase, PfNDI1 (PFI0735c), with closest homology to Ndi1p, was found in the *P. falciparum* genome database [13]. Similarly, Nde1p and Nde2p do not have counterparts in mammalian cells, which utilize redox shuttle mechanisms to couple cytosolic NADH oxidation to NADH dedydrogenase (complex I) in the matrix side. To test whether PfNDI1 expression in yeast confers artemisinin's sensitivity, PfNDI1 was amplified from a cDNA pool of *P. falciparum*. As expected, expression of PfNDI1 in *ndi1Δ* background partially restored its sensitivity to artemisinin (Figure 2C). This indicates that PfNDI1 expressed in yeast interacts with artemisinin in a similar fashion to Ndi1p.

The third artemisinin-resistant strain is *sip5Δ* (Figure 2A). Little is known about Sip5p except that it biochemically interacts with Snf1 protein kinase and Reg1/Glc7 protein phosphatase [14], both of which are involved in the regulation of alternative carbon source utilization and respiration. Thus, it is possible that *SIP5* somehow indirectly affects energy generation, or, more specifically, mitochondrial respiration. To test the hypothesis that respiration control is involved in artemisinin resistance, mutants of seven other components involved in carbon-source regulation and respiration [15] were examined. Most of them are variably resistant to the action of artemisinin (see Materials and Methods).

The epistatic relationship of *SIP5* with *NDI1* or *NDE1* was also explored. Overexpression of *SIP5* in the control strain, *ndi1Δ* or *nde1Δ* did not have any effect on sensitivity or resistance to artemisinin. In contrast, when either *NDI1* or *NDE1* is overexpressed in *sip5Δ* background, yeast still exhibit hypersensitivity to artemisinin. These findings suggest that *sip5Δ* affects artemisinin resistance indirectly by acting on the electron transport chain. The identification of these three genes that are directly or indirectly involved in respiration reinforced our previous interpretation that artemisinin acts through mitochondria.

Because the electron transport chain is involved in the action of artemisinin, we asked further whether other components downstream of NADH dehydrogenase interact with artemisinin. We have checked available deletion mutants (25 strains altogether) of other known genes in the electron transport chain for possible artemisinin resistance. Although some of these strains cannot grow on nonfermentable media, of those that can, none appears to be resistant to the action of artemisinin (see Materials and Methods). Although it is likely some of these mutants have impaired electron transport activity, the fact that all 25 deletion strains do not manifest either artemisinin hypersensitivity or artemisinin resistance (compared to the wild-type strain) implies that not all components of the pathway interact with artemisinin.

### Artemisinin's Action Generates Reactive Oxygen Species

Generation of oxidative stress is believed to be involved in the antiparasitic effects of artemisinin [16]. Figure 3A shows that the levels of reactive oxygen species (ROS) produced in three strains after treatment with artemisinin positively correlated with their levels of sensitivity to artemisinin. We thus investigated whether the artemisinin-resistant mutants that we isolated were cross-resistant to other oxidative agents. Unexpectedly, these mutants did not show resistance to peroxide or paraquat, and, if anything, manifested hypersensitivity to these oxidative agents (Figure 3B). On the other hand, although *sod1Δ* and *sod2Δ* mutants display increased sensitivity to oxidative stress, neither of them exhibited more sensitivity to artemisinin than the wild type (result not shown). This result, combined with the observation that yeast cells are unaffected by artemisinin in YPD, suggests that artemisinin is not simply a promiscuous oxidant. Instead, we suggest that artemisinin may have local effects on the mitochondria.

**Figure 3:**
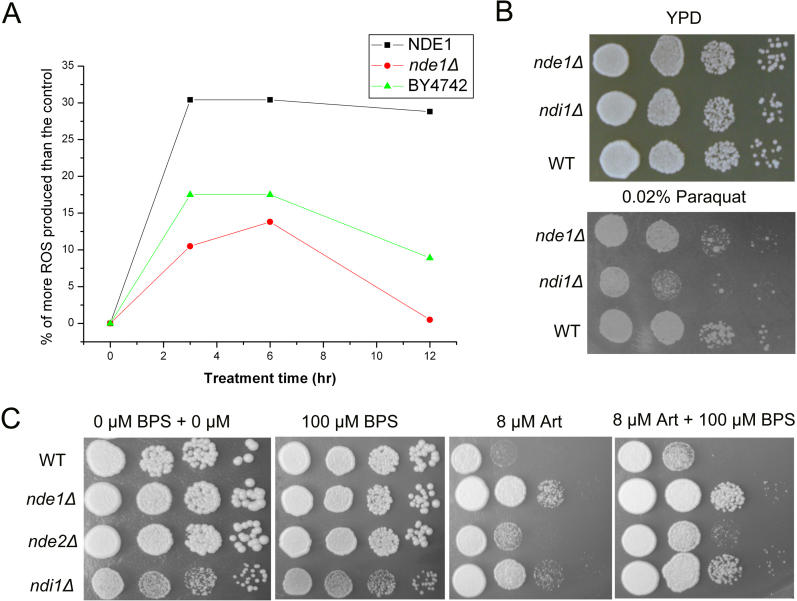
Artemisinin Generates ROS in Yeast When applicable, 8 μM artemisinin was used. (A) Artemisinin-resistant strains generate fewer ROS. Yeast untreated with artemisinin was used as the control. The experiment was performed three times with similar results. NDE1 denotes the overexpressor strain of *NDE1* driven by *ADH1* promoter. (B) Isolated artemisinin-resistant strains are not cross-resistant to paraquat or peroxide. Shown here are the wild-type (WT) parental strain (BY4742), *nde1*Δ and *ndi1*Δ on YPD plates without or with 0.02% paraquat. (C) Iron is possibly involved in artemisinin (Art) activation. Addition of BPS to the medium reduces yeast's sensitivity to artemisinin, whereas BPS alone does not enhance general yeast survival on drug-free plates. We did not use a higher amount of BPS to further reduce the iron level because a severe reduction in iron dramatically affects yeast growth on YPG.

Because activation of artemisinin is known to depend upon the cleavage of a peroxide bridge via an iron-dependent mechanism [17], we investigated the interaction between iron and artemisinin in yeast. When bathophenanthrolinedisulfonic acid (BPS), an iron chelator, was added into YPG media together with 8 μM artemisinin, the inhibitory effect of artemisinin on growth was attenuated (Figure 3C). In a separate series of experiments to determine whether extra iron could aggravate the effect of artemisinin, we added between 1 and 100 μM Fe^2+^ or Fe^3+^ to the plates. At these concentrations iron-enhanced toxicity was not observed. In addition, incubation with iron before artemisinin exposure does not exacerbate yeast sensitivity to artemisinin. These results suggest iron plays an important role in the activation of artemisinin, but additional iron above a certain threshold level has no further effect. This is in agreement with the report that artemisinin can also effectively kill parasites at stages that lack haemozoin [18].

## Discussion

In this report we developed a sensitive yeast model to analyze the action of artemisinin. We demonstrated that artemisinin is able to depolarize the mitochondrial membrane and that increased electron transport activity increases the sensitivity of the cell to artemisinin. Further, we showed artemisinin produces an increase in ROS by a mechanism that differs from that of general oxidants.

Although low amounts of artemisinin affect yeast growth only in nonfermentable media, in both YPG(E) and YPD media artemisinin depolarizes the mitochondrial membrane. The fact that YPD slightly attenuates the effect of artemisinin in depolarizing mitochondrial membrane potential is possibly due to the partial repression of the activity of respiration enzymes (including *NDI1* and *NDE1*) by glucose [19]. Likewise, mutations that indirectly affect respiration will possibly also affect artemisinin sensitivity. The positive correlation observed between respiration activity and sensitivity to artemisinin suggests that the electron transport chain acts to stimulate the activity of artemisinin. As such, the mitochondrial electron transport chain per se does not seem to be the primary target of artemisinin; instead, the electron transport chain seems to play a role in activating artemisinin. The activated artemisinin may then locally depolarize the mitochondrial membrane, impeding many functions dependent upon its potential. Although the disruption of mitochondrial function does not dramatically affect yeast growth in YPD media, for *P. falciparum,* loss of membrane potential will additionally affect pyrimidine biosynthesis [20], a key metabolic process for the survival of the parasite. This proposed mode of action for artemisinin differs from that of atovaquone, a broad-spectrum antiparasitic drug, which has been shown to inhibit complex III of the electron transport chain and, consequently, collapse mitochondrial membrane potential and kill *P. falciparum* [21].

Our findings indicate that iron is involved in the action of artemisinin. Mitochondria are well known to be a rich source of transition metals, including iron and copper. Conceivably, an active electron transport chain provides the electron source to Fe-S in catalyzing artemisinin and produces carbon-centered free radicals. Although we cannot exclude the possibility that other components in the electron transporting chains may interact with artemisinin, our current findings suggest that limited components, including NADH dehydrogenases, are involved in this process.

Distinctive ultrastructural changes have been noted in artemisinin-treated *P. falciparum,* including marked swelling of the mitochondria, followed by the appearance of electron-dense chromatin materials in the nuclei, clumping of ribosomes, nuclear membrane blebbing, and segregation of the nucleoplasm [22–26]. At high artemisinin concentration, even mammalian cells partially lose their mitochondria function [27,28]. These observations are consistent with a primary role of mitochondria in the action of artemisinin, whereas other physiological changes are probably secondary. Not surprisingly, *P. falciparum* has a functional mitochondrial electron transport system and an oxygen-requiring system that is necessary for growth and survival [29–31]. The relative insensitivity of mammalian cells to artemisinins may be due to the fundamental structural difference of their electron transport chains; for example, human complex I is composed of at least 43 components whereas its counterparts in yeast and *P. falciparum* are much simpler (one polypeptide chain). Indeed, a BLAST search with Nde1p or Ndi1p as a query revealed no significant homolog in the human genome. Even more, no significant homologs could be found in available National Center for Biotechnology Information (NCBI) databases across the whole Metazoan kingdom, indicating NADH dehydrogenase is not evolutionarily highly conserved between yeast and Metazoa.

The yeast model provides us with insights into mode of action of artemisinin: It interacts with the electron transport chain, generates local ROS, and causes the depolarization of the mitochondrial membrane. This scheme agrees with what we know about the chemical properties of artemisinin and findings in malaria studies. Although some physiological differences exist between yeast and malarial parasites, the pathway or basic principles that we have derived in our studies of artemisinin in yeast should aid future malaria research. The yeast model that we have developed provides a new way to probe the action of artemisinin. Our findings suggest many future genetic and biochemical analyses of this natural, powerful, and mysterious drug.

## Materials and Methods

### Yeast growth and library screen.

Standard yeast media and growth conditions were used. The yeast deletion library used is a collection of 4,757 homozygous diploid *S. cerevisae* strains (BY4743: *MAT*a/*MAT*á his3Δ1/*his3*Δ1 *leu2*Δ0/*leu2*Δ0 *lys2*Δ0/+ *met15*Δ0/+ *ura3*Δ0/*ura3*Δ0) in which each strain has a single open reading frame replaced with the KanMX4 module. We used homozygous diploid strain library for the original screen because haploid strain appears to have a relatively higher rate of screening background, presumably due to spontaneous mutation. An aliquot of the pooled yeast library was plated on YPG (2% glycerol as carbon source) or YPE (3% ethanol as carbon source) agar plates supplemented with 3–5 μM artemisinin. (Altogether about 100,000 total colonies were plated). Artemisinin-resistant colonies were isolated, and serial dilutions were made and spotted on artemisinin plates to confirm the original phenotype. About a dozen relatively more resistant colonies were chosen, and the corresponding genes were identified by bubble PCR or inverse PCR and DNA sequencing analysis. Phenotypes of these mutants were further confirmed in haploid background (BY4742: *MAT*α *his3*Δ1 *leu2*Δ0 *lys2*Δ0 *ura3*Δ0).

### Artemisinin sensitivity/resistance assay.

For growth in liquid, yeast were grown overnight in YPD medium, spun down, washed three times with YPG medium, and diluted to an A_600_ of 0.5. Then, 10 μl was inoculated into YPD or YPG media with or without artemisinin, and A_600_ was measured over time. For growth on agar plates, yeast previously grown in liquid YPD was spotted with 10-fold serial dilutions.

Besides *SIP5,* genes that may be involved in carbon-source utilization were individually checked for artemisinin sensitivity/resistance. These include *GAL83, SIP1, −2, −3, and −4,* and *SNF1* and *−4*. *snf1* cannot grow on YPG. *sip1Δ, sip3Δ*, *sip4Δ,* and *gal83Δ* display artemisinin resistance.

Mutants of genes in the electron transport chain, including *SDH1, −2, −3,* and *−4, COR1, CYT1, QCR2, −6, −7, −8, −9,* and *-10, RIP1, INH1, STF1* and *-2,* and *COX4, −5A, −5B, −6, −7, −8, −9, −12,* and -*13* were individually examined for artemisinin resistance. Among these, *SDH1* and *-3, CYT1, QCR9,* and *COX4, −6, −7,* and *-13* are required for yeast growth on YPG media, precluding analysis of their involvement in artemisinin's action.

### Gene deletion and expression.

Gene replacement was achieved by homologous recombination with *URA3* or *LEU2* as the replacement marker in strain BY4742 background. Gene deletions were verified by PCR. For expression*, SIP5, NDI1, NDE1,* and PfNDI1 were PCR amplified from BY4743 genomic DNA or a cDNA library of *P. falciparum*. Amplified target DNAs were then cloned into an *ADH1*-driven expression vector (p*ADH1*-YES2, a vector modified from pYES2 from Invitrogen (Carlsbad, California, United States), in which the *GAL1* promoter was replaced with an *ADH1* promoter). Yeast transformation was done by the lithium acetate method. Transformed cells were selected on SD-Ura. Sequences for PCR primers used for *SIP5, NDI1, NDE1,* and PfNDI1 are available by request.

### Analysis of ROS production.

Flow cytometric analysis was used to assay the production of free intracellular radicals. Briefly, cells were incubated with dihydrorhodamine 123 for 2 h and then analyzed by a FACS Calibur (Becton Dickinson, San Jose, California, United States) at a low flow rate with excitation and emission settings of 488 and 525–550 nm (filter FL1), respectively.

### Assay of the electrochemical potential.

After treatment in 20 mM HEPES buffer (pH 7.4) containing 50 mM glucose, 1 ml of the cell suspension was incubated with 2 μM Rh123 (rhodamine 123) for 30 min, washed, and then resuspended in 100 μl PBS. Mitochondrial electrochemical potential was expressed as the fluorescence intensity of Rh123, which was read through a FACS Calibur (Becton Dickinson) with excitation at 480 nm and emission at 530 nm.

## Abbreviations

BPS: bathophenanthrolinedisulfonic acid
IC_50_: 50% growth inhibition concentration
ROS: reactive oxygen species
SG: synthetic yeast media with glycerol as the carbon source
YPG(E): yeast media with glycerol or ethanol as the carbon source
YPGE: yeast media with glycerol and ethanol as carbon sources
